# Risk-reducing hysterectomy and bilateral salpingo-oophorectomy in female heterozygotes of pathogenic mismatch repair variants: a Prospective Lynch Syndrome Database report

**DOI:** 10.1038/s41436-020-01029-1

**Published:** 2020-12-01

**Authors:** Mev Dominguez-Valentin, Emma J. Crosbie, Christoph Engel, Stefan Aretz, Finlay Macrae, Ingrid Winship, Gabriel Capella, Huw Thomas, Sigve Nakken, Eivind Hovig, Maartje Nielsen, Rolf H. Sijmons, Lucio Bertario, Bernardo Bonanni, Maria Grazia Tibiletti, Giulia Martina Cavestro, Miriam Mints, Nathan Gluck, Lior Katz, Karl Heinimann, Carlos A. Vaccaro, Kate Green, Fiona Lalloo, James Hill, Wolff Schmiegel, Deepak Vangala, Claudia Perne, Hans-Georg Strauß, Johanna Tecklenburg, Elke Holinski-Feder, Verena Steinke-Lange, Jukka-Pekka Mecklin, John-Paul Plazzer, Marta Pineda, Matilde Navarro, Joan Brunet Vidal, Revital Kariv, Guy Rosner, Tamara Alejandra Piñero, María Laura Gonzalez, Pablo Kalfayan, Neil Ryan, Sanne W. ten Broeke, Mark A. Jenkins, Lone Sunde, Inge Bernstein, John Burn, Marc Greenblatt, Wouter H. de Vos tot Nederveen Cappel, Adriana Della Valle, Francisco Lopez-Koestner, Karin Alvarez, Reinhard Büttner, Heike Görgens, Monika Morak, Stefanie Holzapfel, Robert Hüneburg, Magnus von Knebel Doeberitz, Markus Loeffler, Nils Rahner, Jürgen Weitz, Kirsi Pylvänäinen, Laura Renkonen-Sinisalo, Anna Lepistö, Annika Auranen, John L. Hopper, Aung Ko Win, Robert W. Haile, Noralane M. Lindor, Steven Gallinger, Loïc Le Marchand, Polly A. Newcomb, Jane C. Figueiredo, Stephen N. Thibodeau, Christina Therkildsen, Henrik Okkels, Zohreh Ketabi, Oliver G. Denton, Einar Andreas Rødland, Hans Vasen, Florencia Neffa, Patricia Esperon, Douglas Tjandra, Gabriela Möslein, Julian R. Sampson, D. Gareth Evans, Toni T. Seppälä, Pål Møller

**Affiliations:** 1 Department of Department of Tumor Biology, Institute of Cancer Research, The Norwegian Radium Hospital, Oslo, Norway; 2 Division of Cancer Sciences, Faculty of Biology, Medicine and Health, University of Manchester and St Mary’s Hospital, Manchester, UK; 3 Directorate of Gynaecology, Manchester University NHS Foundation Trust, Manchester, UK; 4 Institute for Medical Informatics, Statistics and Epidemiology, University of Leipzig, Leipzig, Germany; 5 Institute of Human Genetics, University of Bonn, Bonn, Germany; 6 National Center for Hereditary Tumor Syndromes, University Hospital Bonn, Bonn, Germany; 7 Colorectal Medicine and Genetics, The Royal Melbourne Hospital, Melbourne, Australia; 8 Department of Medicine, Melbourne University, Melbourne, Australia; 9 Hereditary Cancer Program, Institut Catal. d’Oncologia-IDIBELL, L’Hospitalet de Llobregat, Barcelona, Spain; 10 St Mark’s Hospital, Department of Surgery and Cancer, Imperial College London, London, UK; 11 Centre for Cancer Cell Reprogramming, Institute of Clinical Medicine, Faculty of Medicine, University of Oslo, Oslo, Norway; 12 Department of Informatics, University of Oslo, Oslo, Norway; 13 Leids Universitair Medisch Centrum, Leiden, Netherlands; 14 Department of Genetics, University of Groningen, University Medical Center Groningen, Groningen, The Netherlands; 15 IEO, European Institute of Oncology IRCCS, Milan, Italy; 16 Fondazione IRCCS Istituto Nazionale dei Tumori, Milan, Italy; 17 Ospedale di Circolo ASST Settelaghi, Centro di Ricerca tumori eredo-familiari, Università dell’Insubria, Varese, Italy; 18 Gastroenterology and Gastrointestinal Endoscopy Unit, Vita-Salute San Raffaele University, San Raffaele Scientific Institute, Milan, Italy; 19 Department of Women’s and Children’s health, Division of Obstetrics and Gyneacology, Karolinska Institutet, Karolinska University Hospital, Solna, Stockholm Sweden; 20 Tel-Aviv Sourasky Medical Center, Research Center for Digestive Disorders and Liver Diseases, Tel-Aviv, Israel; 21 Department of Gastroenterology, Tel-Aviv Sourasky Medical Center and Sackler Faculty of Medicine, Tel-Aviv University, Tel-Aviv, Israel; 22 High Risk and GI Cancer prevention Clinic, Gastro-Oncology Unit, The Department of Gastroenterology, Sheba Medical Center, Tel-Aviv, Israel; 23 Medical Genetics, Institute for Medical Genetics and Pathology, University Hospital Basel, Basel, Switzerland; 24 Hereditary Cancer Program (PROCANHE) Hospital Italiano de Buenos Aires, Buenos Aires, Argentina; 25 Instituto de Ciencias Básicas y Medicina Experimental (ICBME)-Instituto Universitario (IU)-Hospital, Buenos Aires, Argentina; 26 Manchester Centre for Genomic Medicine, Manchester University Hospitals NHS Foundation Trust, Manchester, UK; 27 Department of Surgery, Manchester University Hospitals NHS Foundation Trust and University of Manchester, Manchester, UK; 28 Department of Medicine, Knappschaftskrankenhaus, Ruhr-University Bochum, Bochum, Germany; 29 Department of Gynaecology, University Clinics, Martin-Luther University, Halle-Wittenberg, Germany; 30 Institute of Human Genetics, Hannover Medical School, Hannover, Germany; 31 Medizinische Klinik und Poliklinik IV, Campus Innenstadt, Klinikum der Universität München, Munich, Germany; 32 MGZ Medical Genetics Center, Munich, Germany; 33 Faculty of Sport and Health Sciences, University of Jyväskylä, Jyväskylä, Finland; 34 Department of Surgery, Central Finland Health Care District, Jyväskylä, Finland; 35 The Royal Melbourne Hospital, Melbourne, Australia; 36 Hereditary Cancer Program, Institut Català d’Oncologia-IDIBELL, L’Hospitalet de Llobregat, Barcelona, Spain; 37 Centre for Epidemiology and Biostatistics, Melbourne School of Population and Global Health, The University of Melbourne, Parkville, VIC Australia; 38 Department of Clinical Genetics, Aalborg University Hospital, Aalborg, Denmark; 39 Department of Biomedicine, Aarhus University, Aarhus, Denmark; 40 Department of Surgical Gastroenterology, Aalborg University Hospital, Aalborg, Denmark; 41 Department of Clinical Medicine, Aalborg University, Aalborg, Denmark; 42 Faculty of Medical Sciences, Newcastle University, Newcastle upon Tyne, UK; 43 University of Vermont, Larner College of Medicine, Burlington, VT 05405 USA; 44 Department of Gastroenterology and Hepatology, Isala Clinics, Zwolle, The Netherlands; 45 Grupo Colaborativo Uruguayo, Investigación de Afecciones Oncológicas Hereditarias (GCU), Hospital Fuerzas Armadas, Montevideo, Uruguay; 46 Lab. Oncología y Genética Molecular, Unidad de coloproctología Clínica Las Condes, Santiago, Chile; 47 Institute of Pathology, University of Cologne, Cologne, Germany; 48 Department of Surgery, Technische Universität Dresden, Dresden, Germany; 49 Department of Internal Medicine I, University Hospital Bonn; National Center for Hereditary Tumor Syndromes, University Hospital Bonn, Bonn, Germany; 50 Department of Applied Tumour Biology, Institute of Pathology, University Hospital Heidelberg, Heidelberg, Germany; 51 Cooperation Unit Applied Tumour Biology, German Cancer Research Center (DKFZ), Heidelberg, Germany; 52 Institute of Human Genetics, Medical School, Heinrich Heine University, Duesseldorf, Germany; 53 Department of Education and Science, Central Finland Health Care District, yväskylä, Finland; 54 Department of Obstetrics and Gynecology and Tays Cancer Centre, Tampere University Hospital and Tampere University, Tampere, Finland; 55 Department of Gastrointestinal Surgery, Helsinki University Central Hospital, Applied Tumour Genomics Research Program, University of Helsinki, Helsinki, Finland; 56 Department of Medicine, Division of Oncology, Stanford Cancer Institute, Stanford University, Stanford, USA; 57 Department of Health Science Research, Mayo Clinic Arizona, Phoenix, AZ USA; 58 Lunenfeld Tanenbaum Research Institute, Mount Sinai Hospital, University of Toronto, Toronto, ON Canada; 59 University of Hawaii Cancer Center, Honolulu, HI USA; 60 Public Health Sciences Division, Fred Hutchinson Cancer Research Center, Seattle, WA USA; 61 Public Health Sciences Division, Fred Hutchinson Cancer Research Center, Seattle, WA USA; 62 Department of Laboratory Medicine and Pathology, Mayo Clinic, Rochester, MN USA; 63 The Danish HNPCC register, Clinical Research Centre, Copenhagen University Hospital, Hvidovre, Denmark; 64 Department of Molecular Diagnostics, Aalborg University Hospital, Aalborg, Denmark; 65 Dept. of Obstetrics and Gynaecology, Copenhagen University Hospital, Rigshospitalet, Denmark; 66 Institute of Medical Genetics, Division of Cancer and Genetics, Cardiff University School of Medicine, Cardiff, UK; 67 Department of Gastroenterology and Hepatology, Leiden University Medical Centre, Leiden, The Netherlands; 68 Colorectal Medicine and Genetics, The Royal Melbourne Hospital, Melborne, Australia; 69 Department of Medicine, Melbourne University, Melborne, Australia; 70 Surgical Center for Hereditary Tumors, Ev. Bethesda Khs Duisburg, University Witten-Herdecke, Herdecke, Germany; 71 Division of Evolution and Genomic Medicine, University of Manchester, Manchester, UK; 72 Manchester Centre for Genomic Medicine, Manchester University NHS Foundation Trust, Manchester Academic Health Science Centre, Manchester, UK; 73 Department of Gastrointestinal Surgery, Helsinki University Central Hospital, University of Helsinki, Helsinki, Finland; 74 Department of Surgical Oncology, Johns Hopkins Hospital, Baltimore, MD USA

## Abstract

**Purpose:**

To determine impact of risk-reducing hysterectomy and bilateral salpingo-oophorectomy (BSO) on gynecological cancer incidence and death in heterozygotes of pathogenic MMR (*path_MMR*) variants.

**Methods:**

The Prospective Lynch Syndrome Database was used to investigate the effects of gynecological risk-reducing surgery (RRS) at different ages.

**Results:**

Risk-reducing hysterectomy at 25 years of age prevents endometrial cancer before 50 years in 15%, 18%, 13%, and 0% of *path_MLH1*, *path_MSH2*, *path_MSH6*, and *path_PMS2* heterozygotes and death in 2%, 2%, 1%, and 0%, respectively. Risk-reducing BSO at 25 years of age prevents ovarian cancer before 50 years in 6%, 11%, 2%, and 0% and death in 1%, 2%, 0%, and 0%, respectively. Risk-reducing hysterectomy at 40 years prevents endometrial cancer by 50 years in 13%, 16%, 11%, and 0% and death in 1%, 2%, 1%, and 0%, respectively. BSO at 40 years prevents ovarian cancer before 50 years in 4%, 8%, 0%, and 0%, and death in 1%, 1%, 0%, and 0%, respectively.

**Conclusion:**

Little benefit is gained by performing RRS before 40 years of age and premenopausal BSO in *path_MSH6* and *path_PMS2* heterozygotes has no measurable benefit for mortality. These findings may aid decision making for women with LS who are considering RRS.

## INTRODUCTION

Lynch syndrome (LS) is a common hereditary cancer predisposition syndrome, present in an estimated 1 in 300 individuals, based on prevalence of the underlying genetic abnormalities in the general population. LS is caused by pathogenic variants in one of four DNA mismatch repair (MMR) genes (*path_MMR*): *path_MLH1*, *path_MSH2, path_MSH6*, and *path_PMS2*, each of which result in different risks for cancers, including colorectal, endometrial, ovarian, stomach, small bowel, bile duct, pancreas, urinary tract, brain, and prostate cancer.^1–5^ In women with LS, gynecological cancers are as common as gastrointestinal cancers. Until recently, clinical guidelines were similar for heterozygotes of all *path_MMR* genetic variants, endometrial cancer prognosis was assumed to be similar in heterozygotes and MMR variant-negative individuals, and the prognosis for ovarian cancer was assumed to be similar to ovarian cancer in *path_BRCA1* heterozygotes. The recent Manchester International Consensus Group publication^6^ described the risk for, and survival after, gynecological cancers in LS by genotype, as initially reported by the Prospective Lynch Syndrome Database (PLSD).^1–4,7^ Later, the PLSD reported findings in an additional independent cohort of *path_MMR* heterozygotes that validated the results from its original cohort and allowed merger of both cohorts to obtain more precise risk estimates and calculation of 5-year and 10-year crude survival after cancer.^2^

Risk-reducing surgery (RRS) including total hysterectomy and bilateral salpingo-oophorectomy (BSO) prevents gynecological cancer in Lynch syndrome.^8^ The Manchester International Consensus Group strongly recommended that risk-reducing hysterectomy and BSO are offered no earlier than 35–40 years of age, following completion of childbearing in *path_MLH1*, *path_MSH2*, and *path_MSH6* heterozygotes but the data supporting such recommendations are not strong, and various practices currently exist. There was insufficient evidence to strongly recommend risk-reducing surgery for *path_PMS2* heterozygotes.^6^

In this report, we determine the impact on cancer incidence and mortality of RRS at different ages in heterozygotes of pathogenic MMR variants.

## MATERIALS AND METHODS

The PLSD is an international, multicenter, prospective observational study without a control group. The PLSD design and its inclusion criteria have been described previously in detail.^1,3,4,9,10^

In brief, *path_MMR* heterozygotes, including probands and their relatives, were recruited for prospective follow-up in each participating center. Genetic variants were assumed to be inherited and were found by genetic testing either prior to, at, or after inclusion for follow-up. Inclusion was from the first prospectively planned and completed colonoscopy, and all recruits had subsequent follow-up of one year or more. Any cancers that were diagnosed before or at the same age as the first prospectively planned and completed colonoscopy were scored as previous cancers. Time to first cancer after inclusion was calculated for each organ or groups of organs. Only heterozygotes with pathogenic variants confirmed as class 4 or 5 (clinically actionable) in the International Society for Gastrointestinal Hereditary Tumors (InSiGHT) database (https://databases.lovd.nl/shared/genes) were included. Each patient was censored at the age at which the last information was available, which might have been a colonoscopy, any other clinical examination, a report from an examination done by others, or information that the patient had died, whichever came last. Observation time was censored at organ removal (therapeutic or prophylactic) when calculating incidences for cancer in specific organs.^1^

### Impact on cancer incidence of risk-reducing hysterectomy and/or BSO by age and gene

The inclusion criteria for calculating the endometrial and ovarian cancer risks were (1) female, (2) heterozygotes with pathogenic (class 4 or 5) MMR variant as classified in the InSiGHT database (http://insight-database.org/), (3) no previous hysterectomy or BSO, and (4) aged 25 to 74 years at start of follow-up. The following information was used for analyses: age at last observation, incident endometrial and/or ovarian cancer, *path_MMR* variant, age at hysterectomy, and age at oophorectomy. In this report, we assume the oophorectomies undertaken were all BSO.

Endometrial and ovarian cancer risks are reported by 5-year age groups. These risks may be considered to represent cancers that would have been prevented if surgery had been undertaken before the ages concerned.

All risks used for calculations and their 95% confidence intervals are derived from our previous publications.^1–4^ Briefly, annual incidence rates (AIRs) by age were calculated in 5-year cohorts from 25 to 75 years of age. Cumulative incidence, denoted by Q, was computed starting at age 25, assuming zero incidence rate before age 25, using the formula Q(age) = Q(age − 1) + [1 − Q(age − 1)] × AIR(age) where AIR(age) is the annual incidence rate as estimated from the corresponding 5-year interval. The observed AIRs and cumulative incidence of endometrial and/or ovarian cancer in the current data set have not been described previously and are now presented here in the Supplementary file.

### Risk of dying from endometrial or ovarian cancer

As in all previous PLSD reports, cancer incidence at 25 years of age (the minimum age from which PLSD collects prospective data) was assumed to be zero. In this report, we provide estimates of the risk of dying following endometrial or ovarian cancer, stratified by MMR gene from 25 to 69 years of age. As displayed at our interactive website (www.plsd.eu), the confidence intervals for these measures are wide for patients with heterozygous *path_MSH6* and *path_PMS2* variants, and the point estimates of risks for patients with these genotypes must be used with caution.

Survival after cancer was estimated by the Kaplan–Meier survival function as crude survival from age at diagnosis until last observation or death. All the AIRs and cumulative incidences are prospectively observed empirical observations, while the survival following endometrial and/or ovarian cancer was calculated as follows: at any given age for cumulative incidences in the tables for endometrial or ovarian cancer separately, we calculated the relative risk for having endometrial or ovarian cancer as the incidence of the one divided by the sum of the two incidences.

Survival after endometrial and/or ovarian cancer was calculated as follows. The following observed factors (with acronyms) were entered into the calculations: risk of endometrial cancer (EC_risk_), risk of ovarian cancer (OC_risk_), risk of ovarian and/or endometrial cancer (ECOC_risk_), survival after endometrial cancer (EC_survival_), and survival after ovarian cancer (OC_survival_). The three former were age-dependent while the two latter were the same for all ages. From the two latter, the difference between the survival for ovarian and endometrial cancer (SURV_diff_) was (EC_survival_ – OC_survival_) = 5%, which was the same for all ages. For each age cohort given in the table, the fraction of endometrial cancer (EC_fraction_) was calculated as the risk for endometrial cancer divided by the sum of the risks for endometrial and ovarian cancer as EC_risk_/(EC_risk_ + OC_risk_). OC survival was lower than EC survival and the survival when ovarian and/or endometrial cancer was scored as an event; the interpolated combined survival indicated in the table was calculated as OC_survival_ + SURV_diff_ *EC_fraction_ for all age groups.

## RESULTS

### Survival after endometrial or ovarian cancer

There were 58, 61, 18, and 4 cases of prospectively observed endometrial cancer included in the survival analyses in *path_MLH1*, *path_MSH2*, *path_MSH6*, and *path_PMS2* heterozygotes, respectively. There were 22, 23, 1, and 1 prospectively detected ovarian cancer cases included in the survival analyses in *path_MLH1*, *path_MSH2*, *path_MSH6*, and *path_PMS2* heterozygotes, respectively. The average for all cases was used to estimate survival for all heterozygotes in this report, but numbers of *path_MSH6* and *path_PMS2* heterozygotes were too low for us to determine whether the average survival pertains to these heterozygotes. The numbers of cases were also too few to permit calculations of survival by the age at which cancer occurred.

**Fig. 1:**
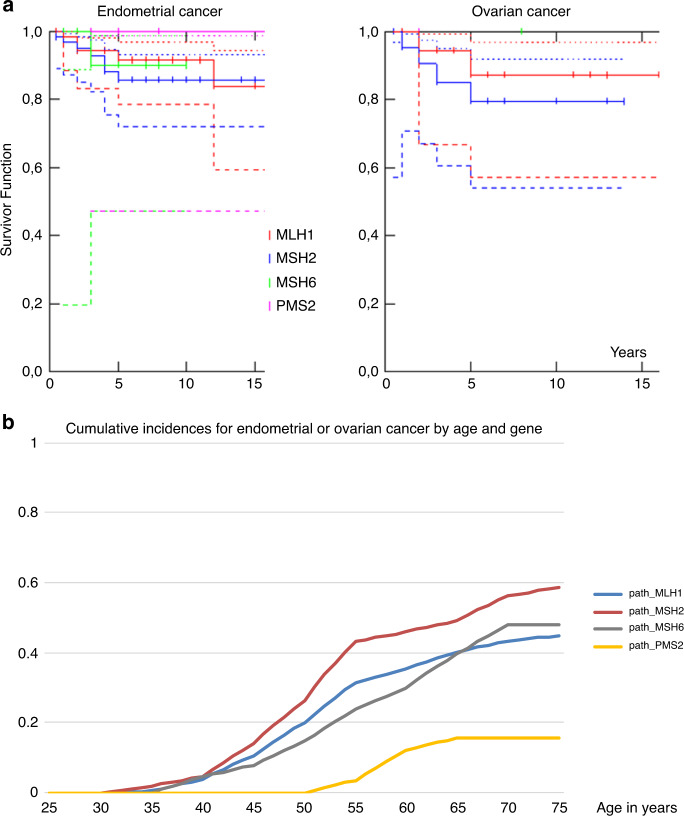
Survival when endometrial or ovarian cancer and cumulative risk by age for endometrial and/or ovarian cancer by pathogenic genetic variant. Whole line indicate point estimates, dotted lines indicate 95% confidence intervals. **a** Survival of endometrial cancer and ovarian cancer by gene. **b** Cumulative incidences of endometrial or ovarian cancer by age and gene.

Estimates of five- and ten-year survival after endometrial or ovarian cancer in LS, but not stratified by gene, have been published previously.^1^ Figure 1 presents survival by gene. As illustrated, there were no significant differences between the genes. After a few early deaths, the curves for both endometrial and ovarian cancer survival flatten out. This is in contrast to the lower reported survival in *path_BRCA1/2*-associated or sporadic ovarian cancer cases for which the survival curve does not flatten out, although deaths beyond 5 years in *BRCA1/2* cases are usually predicted by recurrence before that time.^11^

### Impact on cancer incidence and mortality of risk-reducing hysterectomy and/or BSO by age and gene

Among the heterozygotes included in the last PLSD report^1^ there were 7838 observed female years for *path_MLH1* heterozygotes, 5487 for *path_MSH2*, 1614 for *path_MSH6*, and 862 for *path_PMS2* that met the selection criteria for the current study.

**Fig. 2:**
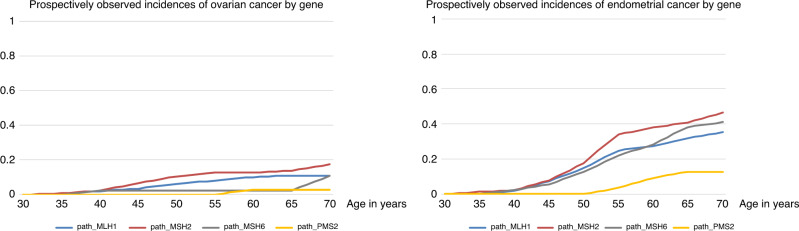
Cumulative incidences of endometrial (to the right) or ovarian (to the left) cancer by age and genetic variant.

In Table 1 and Fig. 2, the risks for endometrial cancer from 25 up to 40, 50, 60, or 70 years of age are given by gene for patients who did not have surgery before each respective age cutoff. Risks from 40, 50, and 60 up to 70 years of age are given to indicate the potential for endometrial cancers to be prevented if hysterectomy is undertaken at these ages. The risks for developing cancer in each 10-year cohort are also given. In Table 2, the corresponding risks for ovarian cancer by age and gene are given. The combined risks for developing and dying from gynecological cancers by age and gene in the absence of risk-reducing hysterectomy and/or BSO are described in Table 3.

**Table 1:** Risks for endometrial cancer in heterozygotes of each *path*_*MMR* gene, 10-year survival, and mortality within 10 years.

Age group	Risk of endometrial cancer diagnosed in the age interval for a heterozygote without cancer at or before entry to the age group				10-year survival	Risk of endometrial cancer diagnosed in the age interval indicated and dying of this within 10 years, for a heterozygote without previous cancer at or before entry to the age group			
	*path_MLH1*	*path_MSH2*	*path_MSH6*	*path_PMS2*		*path_MLH1*	*path_MSH2*	*path_MSH6*	*path_PMS2*
25 to 40 years	2%	2%	2%	0%	89%	0%	0%	0%	0%
**25 to 50 years**	**15%**	**18%**	**13%**	**0%**	**89%**	**2%**	**2%**	**1%**	**0%**
25 to 60 years	27%	38%	28%	9%	89%	3%	4%	3%	1%
25 to 70 years	35%	47%	41%	13%	89%	4%	5%	5%	1%
40 to 70 years	34%	45%	40%	13%	89%	4%	5%	4%	1%
50 to 70 years	24%	35%	33%	13%	89%	3%	4%	4%	1%
60 to 70 years	11%	14%	18%	4%	89%	1%	2%	2%	0%
**40 to 50 years**	**13%**	**16%**	**11%**	**0%**	**89%**	**1%**	**2%**	**1%**	**0%**
50 to 60 years	15%	25%	18%	9%	89%	2%	3%	2%	1%

To the left: the upper four rows indicate risk for endometrial cancer from 25 to 40, 50, 60, or 70 years of age, respectively, if hysterectomy is not undertaken before the ages indicated (i.e., the risk for cancers that could have been prevented by hysterectomy at age 25). The middle three rows indicate the risk for heterozygotes from 40, 50, or 60 years of age, respectively, up to 70 years of age, for cancers that could be prevented by hysterectomy at age 40, 50, or 60 years of age, respectively. The lower two rows indicate the risk for heterozygotes in the age intervals indicated, for cancers that could be prevented by hysterectomy at age 40 or 50, respectively.

**Table 2:** Risks for ovarian cancer in heterozygotes of each *path_MMR* gene, 10-year survival, and mortality within 10 years.

Age group	Risk of ovarian cancer diagnosed in the age interval for a heterozygote without cancer at or before entry to the age group				10-year survival	Risk of ovarian cancer diagnosed in the age interval indicated and dying of this within 10 years, for a heterozygote without previous cancer at or before entry to the age group			
	*path_MLH1*	*path_MSH2*	*path_MSH6*	*path_PMS2*		*path_MLH1*	*path_MSH2*	*path_MSH6*	*path_PMS2*
25 to 40 years	2%	2%	2%	0%	84%	0%	0%	0%	0%
**25 to 50 years**	**6%**	**11%**	**2%**	**0%**	**84%**	**1%**	**2%**	**0%**	**0%**
25 to 60 years	10%	13%	2%	3%	84%	2%	2%	0%	0%
25 to 70 years	11%	17%	11%	3%	84%	2%	3%	2%	0%
40 to 70 years	9%	16%	9%	3%	84%	1%	3%	1%	2%
50 to 70 years	5%	8%	9%	3%	84%	1%	1%	1%	2%
60 to 70 years	1%	6%	9%	0%	84%	0%	1%	1%	1%
**40 to 50 years**	**4%**	**8%**	**0%**	**0%**	**84%**	**1%**	**1%**	**0%**	**0%**
50 to 60 years	4%	2%	0%	3%	84%	1%	0%	0%	1%

To the left: the upper four rows indicate risk for ovarian cancer from 25 to 40, 50, 60, or 70 years of age, respectively, if bilateral salpingo-oophorectomy (BSO) is not undertaken before the ages indicated (i.e., the risk for cancers that could have been prevented by BSO at age 25). The middle three rows indicate the risk for heterozygotes from 40, 50, or 60 years of age, respectively, up to 70 years of age, for cancers that could be prevented by hysterectomy at age 40, 50, or 60 years of age, respectively. The lower two rows indicate the risk for heterozygotes in the age intervals indicated, for cancers that could be prevented by hysterectomy at age 40 or 50, respectively.

**Table 3:** Risks for ovarian or endometrial cancer in heterozygotes of each *path_MMR* gene, 10-year survival, and mortality within 10 years.

Age group	Risk for a healthy heterozygote entering the age group to develop endometrial or ovarian cancer				Combined survival by gene as interpolation of survival as fraction of endometrial and ovarian cancer				Probability of dying from endometrial or ovarian cancer diagnosed in the age group			
	*path_MLH1*	*path_MSH2*	*path_MSH6*	*path_PMS2*	*path_MLH1*	*path_MSH2*	*path_MSH6*	*path_PMS2*	*path_MLH1*	*path_MSH2*	*path_MSH6*	*path_PMS2*
25 to 40 years	4%	5%	5%	0%	87%	87%	87%		1%	1%	1%	0%
25 to 50 years	20%	27%	15%	0%	88%	87%	88%		2%	3%	2%	0%
25 to 60 years	35%	47%	30%	12%	88%	88%	89%	88%	4%	6%	3%	1%
25 to 70 years	43%	58%	48%	16%	88%	88%	88%	88%	5%	7%	6%	2%
40 to 70 years	41%	56%	46%	16%	88%	88%	88%	87%	5%	7%	5%	2%
50 to 70 years	29%	42%	39%	16%	88%	88%	88%	87%	3%	5%	5%	2%
60 to 70 years	12%	20%	26%	4%	89%	88%	87%	87%	1%	2%	3%	1%
40 to 50 years	17%	23%	11%	0%	88%	87%	89%		2%	3%	1%	0%
50 to 60 years	19%	28%	18%	12%	88%	89%	89%	87%	2%	3%	2%	2%

To the left: the upper four rows indicate risk for ovarian cancer from 25 to 40, 50, 60, or 70 years of age, respectively if hysterectomy and bilateral salpingo-oophorectomy (BSO) are not undertaken before the ages indicated (i.e., the risk for cancers that could have been prevented by hysterectomy and BSO at age 25). The middle three rows indicate the risk for heterozygotes from 40, 50, or 60 years of age, respectively, up to 70 years of age, for cancers that could be prevented by hysterectomy and BSO at age 40, 50, or 60 years of age, respectively. The lower two rows indicate the risk for heterozygotes in the age intervals indicated, for cancers that could be prevented by hysterectomy and BSO at age 40 or 50, respectively.

If risk-reducing hysterectomy were performed at 25 years of age, endometrial cancer before 50 years would be prevented in 15%, 18%, 13%, and 0%, in patients with heterozygous *path_MLH1*, *path_MSH2*, *path_MSH6*, and *path_PMS2* variants, respectively, and death in 2%, 2%, 1%, and 0%. If risk-reducing BSO had been performed at 25 years of age, this would have prevented the observed risks of ovarian cancer to age 50 years of 6%, 11%, 2%, and 0% in patients with heterozygous *path_MLH1*, *path_MSH2*, *path_MSH6*, and *path_PMS2* variants, respectively. Correspondingly, the observed ovarian cancer death risks by age 50 years of 1%, 2%, 0%, and 0% would have been prevented (Tables 1 and 2).

Risk-reducing hysterectomy at 40 years of age was estimated to prevent endometrial cancer by 50 years in 13%, 16%, 11%, and 0% of patients and death in 1%, 2%, 1%, and 0% for *path_MLH1*, *path_MSH2*, *path_MSH6*, and *path_PMS2* heterozygotes, respectively. Similarly, BSO carried out at 40 years of age was estimated to prevent ovarian cancer before 50 years of age in 4%, 8%, 0%, and 0%, and to prevent death before 50 years in 1%, 1%, 0%, and 0%, respectively.

## DISCUSSION

In this report, we describe the consequences of RRS by age and gene on incident gynecological cancer risk and associated deaths using observational data from the PLSD from 25 to 69 years of age for different intervention and observation endpoints. Our intention is to empower individual *path_MMR* heterozygotes to make an informed choice regarding whether or not to have risk-reducing gynecological surgery, and the optimal timing for this.

The results in Tables 1, 2, and 3 showing the consequences of having or not having hysterectomy and/or BSO at various ages demonstrate for *path_MLH1*, *path_MSH2*, and *path_MSH6* heterozygotes a small cumulative cancer risk (2%) up to 40 years of age, and a more substantial risk (1.1% to 2.5% annual incidence)^1^ for endometrial cancer from 40 years of age onward. For these patients, the cumulative risk for ovarian cancer from 25 to 50 years is 6%, 11%, and 2% respectively, which combined with the average mortality, which is substantially lower than in *BRCA1/2*-associated or sporadic ovarian cancer, indicate a risk of dying from a premenopausal ovarian cancer to be 1%, 2%, and 0%, respectively. There is also a risk for postmenopausal ovarian cancer. Interpretation of estimates for RSS-associated endometrial and ovarian cancer survival benefit indicates that the absolute reduction in risk of cancer death achieved by very early RRS is small. Performing RRS on 25-year-olds instead of 40-year-olds yields incidence benefits of 0–3%, depending on the *path_MMR* gene, for endometrial and ovarian cancer mortality. These risk estimates are the best we currently have for informing the outcome of premenopausal BSO.

For *path_PMS2* heterozygotes, there is no demonstrable risk for premenopausal endometrial or ovarian cancer, and therefore no argument for considering premenopausal RRS. Similarly, no increase in risk for postmenopausal ovarian cancer has been demonstrated in *path_PMS2* heterozygotes and therefore there is no argument to consider postmenopausal BSO in this group differently from the general population.^1,12^

The cumulative risks for endometrial cancer in *path_MLH1*, *path_MSH2*, and *path_MSH6* heterozygotes illustrated in Fig. 1 may give the impression that the annual incidence rates are substantially lower at older ages. As seen in Table 1, however, this is not so: the risk for endometrial cancer remains high at older ages. Figure 1 shows the typical S-shaped curves generated by conditional probabilities when risk initially increases with age. Because there are fewer older female heterozygotes who have not had endometrial cancer (or hysterectomy), residual risk at older ages results in a lower number of cancer cases than at younger ages, despite high annual incidence among older heterozygotes who have not already had cancer. The higher the risk in younger heterozygotes, the more pronounced this effect will be. Similarly, the combined cumulative incidence by age for endometrial or ovarian cancer as seen in Table 3 is slightly lower than the sum of the two as presented in Tables 1 and 2, because standard treatment of the one removes the risk of having the other at a later time.

While Tables 1 and 2 indicate risks for cancer and survival by age and gene at entry into each age group, any patient may input her actual age and specific genetic variant into the interactive website www.plsd.eu, which will return the risk for cancer in any organ from her current age to any future selected age. From this, one may calculate the risk of dying from that cancer using our previously published survival estimates for LS patients who are affected by that cancer. The figures derived are point estimates and should be interpreted with appropriate caution.

Daily intake of acetyl-salicylic acid (aspirin) has been demonstrated to reduce colon cancer risk in heterozygotes for path_MMR variants by about 50%.^12^ A recent study also demonstrates a reduction in endometrial cancer incidence in heterozygotes for path_MMR variants taking acetyl-salicylic acid.^13^ The results in both of these reports were not stratified by MMR gene or age. The reduced cancer risk was a long-term effect and did not achieve statistical significance for endometrial cancer alone.

This report calculates the impact of RRS on gynecological cancer risk in path_MMR heterozygotes according to age and affected MMR gene, and reports an estimate of a survival benefit in terms of deaths that are actually prevented by RRS. Our calculations are based on the largest international LS database in the world, reporting 15,800 prospective observation years for female *path_MMR* heterozygotes. The prospective registration of incident cancers and associated deaths minimizes ascertainment bias.

There are some limitations to the current study. Low number of patients with *path_MSH6* and *path_PMS2* variants may reflect that they are infrequently identified by the Amsterdam or Bethesda criteria and are infrequently subjected to genetic testing.^14^ With the advent of universal screening of colorectal and endometrial cancers for LS, this situation is likely to change.^6^ We restricted our analysis to report the prospectively observed endometrial and ovarian cancer incidence and survival in women who had not had prophylactic RRS to provide a robust analysis of cancer risk and associated deaths using observational data from the PLSD. We have not investigated for endometrial or ovarian cancer after RRS. When considering survival, it must be remembered that the results presented here were obtained prior to use of immunotherapy for microsatellite unstable tumors: future treatment modalities may further improve the survival, which is already much better than in sporadic or *BRCA*-associated ovarian cancer. Improved imaging and liquid biopsy may make early diagnosis and treatment more effective in future. We have assumed that all bilateral oophorectomies were BSO because type of RRS was not included in our data call.

There is a time-trend bias in the uptake of risk-reducing hysterectomy and BSO: older women may not have had the same option of early risk-reducing surgery that is advocated and available today (and they may not have known they were at risk when they were younger) and the uptake among older women may not be representative of what younger heterozygotes choose today. Because of the inherent time-trend bias, from which no statistical procedures can escape, we considered it inappropriate to investigate the reported uptake of these interventions using more sophisticated statistical methods.

The offer of RRS is currently recommended for women with *path_MMR* variants no earlier than 35–40 years of age^6^ (also see Seppala et al.,^7^ patient 2286). Our intention is to empower individual *path_MMR* heterozygotes to make an informed choice. We do not make management recommendations; rather, we promote personal choice for each *path_MMR* heterozygote based on current data. Since the figures derived are point estimates and should be interpreted with appropriate caution, the use of this information in decision making should be discussed with appropriately trained health-care professionals.

### Conclusions

Our findings may be useful when disclosing results of genetic testing for *path_MMR* variants, since female heterozygotes have to decide which health-care options to select to manage their gynecological cancer risks. Clinical guideline recommendations should now be updated to take account of empirically observed risks for endometrial or ovarian cancers in *path_MMR* heterozygotes by age and gene.

## Supplementary information

Supplementary Information

## Data Availability

The data sets used and/or analyzed during the current study are available from the corresponding author on reasonable request. We have published a website (www.lscarisk.org) on which cancer risks for all published data can be reviewed and calculated in graphic form.
