# Supplementary material for: Risk-reducing hysterectomy and bilateral salpingo-oophorectomy in female heterozygotes of pathogenic mismatch repair variants: a Prospective Lynch Syndrome Database report
Source: Genet Med. 2020 Dec 1;23(4):705–12. doi: 10.1038/s41436-020-01029-1 (PMC8026395; doi:10.1038/s41436-020-01029-1)
Supplement: Supplementary file 1 — Supplementary Information [file 41436_2020_1029_MOESM1_ESM.pdf]

Annual incidence rates observed for first endometrial and/or ovarian cancer scored as event. each carrier scored once only if cancer observed. 25yrs indicates number observation years in carriers aged 25-29 25-29 years. 25ca indicates number of events observed in carriers aged 25-29

**Group**	**25yrs**	**25ca**	**AIR25**	**30yrs**	**30ca**	**AIR30**	**35yrs**	**35ca**	**AIR35**	**40yrs**	**40ca**	**AIR40**	**45yrs**	**45ca**	**AIR45**	**50yrs**	**50ca**	**AIR50**	**55yrs**	**55ca**	**AIR55**	**60yrs**	**60ca**	**AIR60**	**65yrs**	**65ca**	**AIR65**	**70yrs**	**70ca**	**AIR70**	**75yrs**	**75ca**	**AIR75**	**SUM_CA**	**SUM_YRS**
Path_MLH1	716	0	0	1049	1	0.001	1326	9	0.0068	1382	20	0.0145	1174	26	0.0221	848	26	0.0307	579	6	0.0104	431	7	0.0162	231	2	0.0087	86	0	0	9	0	0	97	7831
Path_MSH2	570	0	0	768	3	0.0039	946	5	0.0053	956	20	0.0209	784	25	0.0319	528	27	0.0511	389	5	0.0129	310	4	0.0129	179	4	0.0223	67	0	0	4	0	0	93	5501
Path_MSH6	73	0	0	137	0	0	217	2	0.0092	287	2	0.007	261	4	0.0153	224	5	0.0223	176	3	0.017	137	4	0.0292	101	3	0.0297	38	0	0	2	0	0	23	1653
Path_PMS2	34	0	0	41	0	0	77	0	0	92	0	0	115	0	0	140	1	0.0071	160	3	0.0188	128	1	0.0078	74	0	0	28	0	0	0	0	0	5	889
